# Biodetoxification Using Intravenous Lipid Emulsion, a Rescue Therapy in Life-Threatening Quetiapine and Venlafaxine Poisoning: A Case Report

**DOI:** 10.3390/toxics11110917

**Published:** 2023-11-09

**Authors:** Cristian Cobilinschi, Liliana Mirea, Cosmin-Andrei Andrei, Raluca Ungureanu, Ana-Maria Cotae, Oana Avram, Sebastian Isac, Ioana Marina Grințescu, Radu Țincu

**Affiliations:** 1 Department of Anesthesiology and Intensive Care II, Carol Davila University of Medicine, and Pharmacy, 050474 Bucharest, Romania; cristian.cobilinschi@umfcd.ro (C.C.);; 2 Department of Anesthesiology and Intensive Care, Clinical Emergency Hospital Bucharest, 014461 Bucharest, Romania; 3 Department of Clinical Toxicology, Carol Davila University of Medicine, and Pharmacy, 050474 Bucharest, Romaniar_tincu@yahoo.com (R.Ț.); 4 Department of Anesthesiology and Intensive Care Toxicology, Clinical Emergency Hospital Bucharest, 014461 Bucharest, Romania; 5 Department of Physiology, Carol Davila University of Medicine, and Pharmacy, 050474 Bucharest, Romania; 6 Department of Anesthesiology and Intensive Care, Fundeni Clinical Institute, 022328 Bucharest, Romania

**Keywords:** intravenous lipid emulsion, antidote, venlafaxine, quetiapine, suicidal attempt, cardiotoxicity, neurotoxicity, intoxication

## Abstract

The administration of intravenous lipid emulsion (ILE) is a proven antidote used to reverse local anesthetic-related systemic toxicity. Although the capacity of ILE to generate blood tissue partitioning of lipophilic drugs has been previously demonstrated, a clear recommendation for its use as an antidote for other lipophilic drugs is still under debate. Venlafaxine (an antidepressant acting as a serotonin–norepinephrine reuptake inhibitor (SNRI)) and quetiapine (a second-generation atypical antipsychotic) are widely used in the treatment of psychotic disorders. Both are lipophilic drugs known to induce cardiotoxicity and central nervous depression. We report the case of a 33-year-old man with a medical history of schizoaffective disorder who was admitted to the emergency department (ED) after having been found unconscious due to a voluntary ingestion of 12 g of quetiapine and 4.5 g of venlafaxine. Initial assessment revealed a cardiorespiratory stable patient but unresponsive with a GCS of 4 (M2 E1 V1). In the ED, he was intubated, and gastric lavage was performed. Immediately after the admission to the intensive care unit (ICU), his condition quickly deteriorated, developing cardiovascular collapse refractory to crystalloids and vasopressor infusion. Junctional bradycardia occurred, followed by spontaneous conversion to sinus rhythm. Subsequently, frequent ventricular extrasystoles, as well as patterns of bigeminy, trigeminy, and even episodes of non-sustained ventricular tachycardia, occurred. Additionally, generalized tonic–clonic seizures were observed. Alongside supportive therapy, antiarrhythmic and anticonvulsant therapy, intravenous lipid emulsion bolus, and continuous infusion were administered. His condition progressively improved over the following hours, and 24 h later, he was tapered off the vasopressor. On day 2, the patient repeated the cardiovascular collapse and a second dose of ILE was administered. Over the next few days, the patient’s clinical condition improved, and he was successfully weaned off ventilator and vasopressor support. ILE has the potential to become a form of rescue therapy in cases of severe lipophilic drug poisoning and should be considered a viable treatment for severe cardiovascular instability that is refractory to supportive therapy.

## 1. Introduction

Suicide by drug overdose can be precipitated by a variety of factors, such as psychiatric disorders like depression, anxiety, bipolar disorder, and schizophrenia, among others. Typically, venlafaxine and quetiapine are prescribed to treat these types of disorders.

Quetiapine is an antipsychotic drug belonging to the class of dibenzothiazepine. Low to moderate antagonist activity at multiple neurotransmitter receptor sites is described, such as serotonergic 5-hydroxytryptamine (5-HT2A) receptors, dopaminergic (D1 and D2) receptors, histaminergic (H1) receptors, adrenergic α1 and α2 receptors, and partial agonist activity at 5-HT1A receptors [1]. In a 5-year retrospective analysis of 945 cases diagnosed with acute quetiapine overdose, the main clinical manifestations were drowsiness (76%), coma (10%), seizures (2%), tachycardia (56%), hypotension (18%), and respiratory depression (5%), complications that were more commonly compared with overdose of all other antipsychotic agents [2].

Venlafaxine is a phenethylamine derivative, a selective serotonin and norepinephrine reuptake inhibitor (SNRI). It acts by raising neurotransmitter levels in the brain, which can enhance mood and alleviate symptoms of depression and anxiety [3]. Venlafaxine has been reported to be notably more toxic than selective serotonin reuptake inhibitors (SSRIs) [4]. Clinical manifestations of an overdose of venlafaxine include nausea, vomiting, seizures, agitation, confusion, and changes in blood pressure or heart rate. In critical cases, the overdose may lead to coma, acute respiratory failure, or cardiac arrest. It is also associated with a high prevalence of cardiovascular adverse events. When cardiotoxicity arises, it can manifest itself in a serious manner, as seen in cases of Takotsubo cardiomyopathy, acute coronary syndromes with normal coronary arteries, and malignant arrhythmias. A recent investigation showed that severe cardiotoxicity occurs if the ingestion dose is greater than 8 g, but this is not paramount. Tachycardia and QTc interval prolongation appears to be a dose-dependent effect that may lead to severe cardiac arrhythmias [5,6,7]. If the ingestion dose exceeds 8 g, patients could also manifest other symptoms, including seizures and serotonin toxicity. 

The two drugs are often used to treat mental health conditions. Quetiapine is used to treat schizophrenia, bipolar disorder, and major depressive disorder, whereas venlafaxine is used to treat depression and anxiety disorders. An overdose of either of these drugs can be fatal and necessitates emergency medical intervention. The particular symptoms of an overdose may differ depending on the amount of medication used, as well as individual patient characteristics, such as age and overall health.

Intravenous lipid emulsion (ILE) therapy was initially proposed to address systemic toxicity caused by local anesthetics [8]. Recent case reports have indicated that lipid emulsion infusion may be an effective treatment for non-local anesthetic overdoses involving a diverse range of drugs, including beta-blockers, calcium channel blockers, parasiticides, herbicides, and several psychiatric substances. ILE has been used to treat severe toxicity caused by a variety of lipophilic drugs [9,10]. Even low doses of intravenous lipid emulsion infusion have been reported to be a possibly useful therapy in quetiapine overdose [11]. There are currently no dosage guidelines for drug toxicity other than local anesthetics. 

In this study, we report a case involving a deliberate overdose of quetiapine and venlafaxine, which resulted in a life-threatening situation. However, the patient in question was successfully treated using intravenous lipid emulsion (ILE) therapy.

## 2. Case Report

We report the case of a 33-year-old man with a medical history of schizoaffective disorder, who was admitted to the emergency department (ED) after having been found unconscious due to a voluntary ingestion of 12 g of quetiapine and 4.5 g of venlafaxine. Ingestion was estimated to occur 8 h before presentation. Upon initial assessment, the patient was spontaneous breathing; he was normotensive but comatose with a Glasgow Coma Scale of 4 points (M2 E1 V1) with midline symmetrical dilated pupils and preserved light responses. Considering the patient’s neurological state, he was intubated, and mechanical ventilation was initiated. Gastrointestinal decontamination was promptly performed, consisting of gastric lavage and administration of activated charcoal.

Initial laboratory tests were within normal limits, although the first arterial blood gas analysis revealed elevated lactate of 5.3 m Mol/L. Urine was analyzed by qualitative gas chromatography–mass spectrometry, which was positive for venlafaxine and quetiapine. Additionally, 12 leads electrocardiography (ECG) was recorded without revealing significant changes. The initial QTc interval was assessed at 456 ms.

The patient was admitted to the toxicology intensive care unit, where his condition rapidly deteriorated, developing cardiovascular collapse. The patient’s systolic blood pressure had a downward trend despite volume resuscitation with balanced crystalloids and synthetic colloids. Additionally, vasopressor therapy was initiated with a norepinephrine infusion in doses up to 0.4 μg/kg/min. However, the cardiovascular collapse exhibited resistance to vasopressor intervention and volume resuscitation, resulting in an ongoing decline in blood pressure. Subsequently, the occurrence of junctional bradycardia was observed, which was followed by the spontaneous restoration to sinus rhythm. Subsequently, frequent ventricular extrasystoles, as well as patterns of bigeminy, trigeminy, and even episodes of nonsustained ventricular tachycardia, occurred. In addition, the patient also exhibited generalized tonic–clonic seizures.

A repeated ECG revealed a prolonged QTc of 510 ms. A transthoracic echocardiography exam showed a hyperdynamic left ventricle without abnormalities in wall motion. No right ventricle (RV) dysfunction was registered.

Alongside supportive therapy, antiarrhythmic and anticonvulsant therapy were initiated. Due to severe cardiotoxicity, lipid emulsion 20% (Intralipid^®^ 20%, Fresenius Kabi, Bucharest, Romania) was administered as an intravenous bolus: 1.5 mL/kg over 1 min~100 mL. This was followed by a 0.1 mL/kg/min (approximately 400 mL/h) 2 h continuous infusion. Intravenous magnesium sulphate was administered 18 h post-ingestion when the QTc was 510 ms. The patient’s condition progressively improved over the following hours, and he was weaned off the vasopressor within 90 min of ILE therapy initiation (Table 1). 

On the second ICU day (55 h after ingestion), the patient became hypotensive, requiring vasopressor therapy with noradrenaline in a dose of ~0.2 μg/kg/min. This was followed by sinus bradycardia, which was managed with the help of an atropine bolus. Additionally, a second dose of ILE 1.5 mL/kg was administered.

Over the course of the patient’s stay in the ICU, multiple ECGs were recorded, revealing normalization of QTc after day 6 of ICU. In addition, until day 6 of the patient’s stay in ICU, the urine toxicological exam remained positive for both quetiapine and venlafaxine. 

Hypertriglyceridemia, pancreatitis, and phlebitis, which are common side effects of ILE therapy, were not observed in this case.

Over the next few days, the patient’s clinical condition improved. Sedation was successfully stopped, and afterwards, the patient was weaned from vasopressors and successfully extubated. One week after ingestion, he returned to his neurological baseline. On day 15, the patient was discharged in good clinical condition and referred to a psychiatrist specialist to manage the suicidal attempt.

## 3. Discussion

There is currently no known specific treatment for quetiapine and venlafaxine overdose. In instances where there are more severe symptoms, it is advisable to admit the patient to an intensive care unit where supportive care will be used, such as assuring proper airway, oxygenation, and ventilation, monitoring heart rate and vital signs indicators. Additionally, symptomatic measures are advised. It is not recommended to induce emesis. If performed promptly after intake, gastric lavage may be indicated. Activated charcoal should be used. Due to the large volume of distribution of these drugs, forced diuresis, dialysis, hemoperfusion, and exchange transfusion are unlikely to be of benefit. There are no particular antidotes for venlafaxine or quetiapine overdoses. Intravenous lipid emulsion (ILE) infusion has been reported to be a possibly useful therapy when other interventions have failed [11,12]. Extracorporeal life support should be employed in severe cases of drug-induced cardiotoxicity [13].

Antidepressant poisoning most commonly manifests similarly to serotonin syndrome symptoms, such as high fever, convulsions, mydriasis, and unconsciousness; however, overdose may also cause cardiotoxicity by inhibiting various cardiac ion channels, resulting in sinus bradycardia and QT or QRS prolongation [14]. Atypical antipsychotic overdoses are associated with significantly higher mortality rates. Antipsychotic-induced cardiotoxicity is also caused by alpha-1-adrenergic antagonism, which causes vasoplegia and reflex tachycardia due to antimuscarinic action [15].

The management of patients who have overdosed on both venlafaxine and quetiapine is determined by the degree of overdose and the symptoms that the patient is experiencing. Overdoses of these medications can be fatal.

The immediate release of quetiapine has a linear pharmacokinetic profile with an elimination half-life (t½) of approximately 6–7 h, reaching a peak plasma concentration at 1–1.5 h after ingestion. Additionally, immediate release from the elimination of venlafaxine t½ is 5 ± 2 h. Extended release from the elimination of t½ is 15 ± 6 h. Because plasma concentrations of quetiapine and venlafaxine in this patient were not measured, it is unclear whether the patient’s clearance or metabolism of these drugs was abnormal. Therefore, the second peak of toxicity occurred at 55 h, and it is not clear if it was related to the extended released form of these drugs. This could have been something associated with the late occurrence of cardiotoxicity.

The utilization of ILE therapy has been well recognized as an effective approach to managing local anesthetics systemic toxicity (LAST). Professional bodies such as the American Society of Regional Anesthesia (ASRA) endorse the use of intravenous lipid emulsion (ILE) therapy, referred to as lipid resuscitation therapy (LRT), as the recommended treatment for LAST [16]. Starting with the 2015 American Heart Association guidelines for “Special Circumstances of Resuscitation”, the use of ILE therapy is recommended as a supplementary approach to advanced cardiac life support techniques in cases of suspected cardiac arrest produced by local anesthetic systemic toxicity (LAST) [17].

Additionally, ILE therapy has been employed to treat severe toxicity caused by a variety of lipophilic drugs [9,10]. The mechanism through which lipophilic drug toxicity is treated using ILE therapy is most likely a complex one [12,18]. “The lipid sink theory” hypothesizes that a lipid emulsion traps the drug in the intravascular compartment, preventing it from reaching the peripheral tissues and organs. The reduction in toxicity is achieved by decreasing the concentration of the toxin at the site of its effect. In addition to scavenging, both animal and human models have demonstrated the existence of cardiotonic and postconditioning effects resulting from lipid infusion. Lipids have a direct impact on enhancing cardiac contractility, leading to an improvement in cardiac output and an increase in preload via direct volume expansion [19,20]. However, a growing body of evidence supports the contribution of other mechanisms, such as providing cardiomyocytes with enough free fatty acids used as an alternate energy source and generating a direct positive inotropic effect. Other mechanisms include calcium and sodium channel modulation and vasoplegia reduction via endothelial nitric oxide synthase inhibition [21,22,23]. 

In 2016, the literature was analyzed by a collaborative workgroup, which subsequently formulated clinical guidance regarding the utilization of intravenous lipid emulsion in cases of drug overdose limited only to a few specific situations [24]. Numerous practical aspects, such as the optimal dose, the optimal administration time frame, and the optimal duration of infusion for clinical efficacy, as well as the threshold dose for adverse effects, are still being debated. Current knowledge substantiates the utilization of ILE exclusively in cases of LAST and cardiac toxicity caused by lipophilic drugs when an imminent risk to the patient’s life and alternative treatments have proved to be inefficacious. The use of ILE as a potential antidote is still in its early stages, and more preclinical investigations, clinical studies, and systematic reporting of its usage in humans are required before any recommendations can be made. There is limited comprehension regarding ILE effectiveness, its mechanism of action, and safety [25].

The primary source of information regarding side effects pertains to the administration of intravenous lipids as a form of parenteral nutrition. These adverse effects encompass allergic reactions, hypertriglyceridemia, pancreatitis, bacteremia, fat embolism, thrombophlebitis with peripheral administration, heart failure, lipoid pneumonia, and acute respiratory distress syndrome (ARDS) [26]. 

Toxicology experts currently recommend a 1.5 mL/kg lean body mass of 20% lipid emulsion bolus followed by a continuous infusion of 0.25 mL/kg/min, with options to repeat the bolus or double the infusion rate for persistent cardiovascular instability [27].

The initial documentation of the successful use of lipid emulsion as an antidote for lipophilic, non-local anesthetic toxicity in a human subject was reported in a case involving a 17-year-old female who ingested a substantial amount of bupropion and lamotrigine, resulting in a severe cardiovascular collapse unresponsive to standard advanced cardiovascular life support. A 20% intravenous lipid emulsion was administered as a rescue intervention in an effort to restore hemodynamics, leading to the normalization of vital signs within one minute [28].

In a case series report, intravenous lipid emulsion was administered for the treatment of various lipophilic drug intoxications, resulting in the amelioration of cardiovascular and neurologic symptoms. The report concluded that ILE treatment is an effective life-saving intervention for lipophilic drug intoxications, particularly in unconscious patients presenting with cardiac and/or neurologic manifestations [9]. 

A case report of a 34-year-old patient with a mixed overdose of multiple lipid-soluble drugs documented effects such as a notable decrease in the degree of consciousness and a severe circulatory collapse that did not respond to treatment with adrenaline, noradrenaline, and vasopressin. Additionally, the patient had significant acidemia. However, the clinical condition of the patient began to improve after receiving a lipid infusion, which resulted in a quick reduction in the need for inotropic and vasopressor support, as well as the correction of acidosis [29]. 

A previous case report also details the effective utilization of ILE therapy for a 61-year-old male patient who purposefully ingested excessive quantities of quetiapine and sertraline. Upon arrival at the emergency department, the patient was assessed with a score of 3, according to the Glasgow Coma Scale, and was hypotensive. Around four hours after consumption, a bolus dose of 1.5 mL/kg of a 20% lipid emulsion was administered, followed by an infusion of 6 mL/kg (400 mL). Within fifteen minutes, a notable improvement in the patient’s level of consciousness was registered, resulting in a Glasgow Coma Scale (GCS) score of 9 [30]. 

Another case report describes a patient with acute quetiapine overdose, leading to circulatory collapse. Attempts to stabilize the patient’s condition with vasopressor/inotropic therapy were ineffective. However, the administration of intravenous lipid emulsion (ILE) successfully restored cardiovascular stability [31].

Table 2 contains data from multiple case reports when intravenous lipid therapy was successfully used for acute intoxication with drugs that included quetiapine and/or venlafaxine. Each case described in the table includes the dosing and timing of ILE therapy and the symptoms for which intravenous lipid therapy was used.

There are a few studies that describe the effect of lower doses of ILE therapy, particularly in children. One case report described the successful treatment of quetiapine and citalopram overdose with the recommended bolus dosing, followed by a lower infusion dose of 0.025 mL/kg/min over 1 h [11]. Another case report illustrated the successful treatment of an overdose of quetiapine and fluvoxamine with a 1 mL/kg lipid emulsion bolus followed by a 0.05 mL/kg/min infusion for two hours [39].

The dosing regimen employed in our case was comparable to that utilized by other researchers. This approach resulted in a notable enhancement in clinical outcomes, prompting the decision to prolong the treatment duration for an additional two hours and to repeat the bolus dose when signs of cardiotoxicity reappeared. The administered medication did not exhibit any detrimental consequences, such as allergic responses, fat overload syndrome accompanied by hepatosplenomegaly, jaundice, acute pancreatitis, seizures, fat embolism, coagulopathies, or any alterations in laboratory test results.

After the acute problem is resolved, the patient may require psychological evaluation and treatment to address the root causes of the overdose and prevent future suicidal actions. Medication adjustments and/or hospitalization may be required for further evaluation and care of their mental health condition.

It is crucial to remember that the therapy for venlafaxine and quetiapine overdose can be complicated and may vary based on the patient’s individual previous health status. As a result, it is critical to seek immediate medical assistance and consult with a skilled medical practitioner for advice on the best treatment approach.

## 4. Conclusions

This case report emphasizes the effective use of ILE therapy for severe cardiotoxicity induced by SSRI and antipsychotic overdose. We sustain that the potential utilization of lipid rescue therapy could go beyond its current role and function as a treatment for toxicity induced by local anesthetics.

In summary, despite possible adverse effects, lipid emulsion appears to have an evolving role in the management of a patient with a severe, life-threatening overdose of lipid-soluble compounds. It has been successfully used in the treatment of induced cardiotoxicity by severe overdoses with lipophilic drugs, such as quetiapine and venlafaxine. Overall, the use of intravenous lipid emulsion infusion for venlafaxine and quetiapine overdose needs further investigation. It may be considered as part of a comprehensive treatment plan, especially in those patients with severe cardiotoxicity refractory to maximum conventional therapy.

It should be noted, however, that the use of ILE infusion in drug overdose is still a relatively new and experimental therapy. Its safety and efficacy have not been thoroughly demonstrated. Therefore, it should only be used when the advantages outweigh the hazards and only under the supervision of a skilled medical expert.

## Figures and Tables

**Table 1:** Timeframe in hours following ingestion and clinical and paraclinical markers.

Timeframe (hours following ingestion)	0	12	14	16	18	20	22	24	48	55	72	96	120
Lactate (mmol/L)		5.3	4.3	3.6	4.7	3.5	3.3	3.1	2.2	3.6	2.0	1.5	1
Noradrenaline (mcg/kg/min)				0.25	0.4	0.14	0	0	0	0.2	0.13	0	0
QTc (ms)		456		510				503			480		440
ILE therapy					*					**			

* Intralipid 20%^®^ 1.5 mL/kg—100 mL intravenous bolus over 1 min, followed by a 0.1 mL/kg/min 2-h continuous infusion. ** Intralipid 20%^®^ 1.5 mL/kg—100 mL intravenous bolus over 1 min.

**Table 2:** Case reports on acute intoxications that included quetiapine and/or venlafaxine, in which intravenous lipid emulsion therapy was used.

Reference	Drug	Symptoms	ILE Dose	Timing ILE Therapy	Clinical Outcome
Finn et al. [30]	Quetiapine and sertraline	GCS 3 points	20% lipid emulsion IV bolus dose of 1.5 mL/kg (100 mL) followed by an infusion of 6 mL/kg (400 mL)	4 h after ingestion	Rapid and sustained rise in consciousness (GCS 9) occurred simultaneously with ILE therapy
Bartos and Knudsen [31]	Quetiapine	Cardiovascular collapse refractory to vasopressor treatment and volume resuscitation	20% lipid emulsion IV bolus dose of 170 mL followed by an infusion of 500 mL 20% lipid emulsion run over 1 h		Within the first hour, vasopressor requirement decreased and no further boluses of either epinephrine or phenylephrine were required
Engin et al. [32]	Quetiapine	Depressed consciousness, tachycardia, and hypotension	Two 1.5-mL/kg 20% lipid emulsion IV bolus doses 15 min apart with no infusion drip	One hour after admission to the ICU, ILE therapy was considered to prevent further complications	Symptoms improved
Cevik et al. [9]	Quetiapine	Hypotension and sinus tachycardia	100 mL 20% lipid emulsion IV bolus, followed by 30 mL kg/h infusion over 2 h (total dose of 3580 mL)		Hypotension and tachycardia regressed two hours after ILE treatment
Hieger et al. [33]	Quetiapine	Status epilepticus and cardiovascular collapse	1.5-mL/kg 20% lipid emulsion IV bolus over 5 min, then a drip at 0.25 mL/kg/min (total dose 2000 mL)	11 h after ingestion	Norepinephrine was discontinued
Harvey et al. [34]	Mixed overdose, including Quetiapine and Amitriptyline	Hemodynamic instability with prolonged QRS and QTc	100 mL 20% lipid emulsion IV bolus over 1 min followed by a further 400 mL over 30 min		Hemodynamics improved, QRS and QTc normalized
Purg et al. [11]	Quetiapine, Citalopram, Bromazepam	Life-threatening arrhythmia (prolonged QTc followed by PVC and VT with pules) and status epilepticus	1.5 mL/kg (100 mL) 20% lipid emulsion IV bolus over 10 min, followed by an additional 200 mL over the next hours	12 h after admission	After ILE, QTc normalized, and ventricular tachycardia and seizures stopped
De Wit et al. [35]	Venlafaxine	MODS and refractory shock	1.5 mL/kg (120 mL) 20% lipid emulsion IV as a single bolus dose	40 h after presumed ingestion	Following the administration of the intravenous lipid emulsion, there was a notable improvement in the patient’s clinical condition
Dagtekin et al. [36]	Venlafaxine, Lamotrigine, Diazepam	Rigidity, hyperreflexia, and reflex myoclonia	2.5 mL/kg (150 mL) IV bolus of 20% lipid emulsion	8 h after admision in ICU	Symptoms improved after infusion started
Hillyard et al. [37]	Venlafaxine, Zopiclone	A decline in GCS to 3 points	1.5 mL/kg (100 mL) 20% lipid emulsion IV bolus dose followed by a 400 mL infusion over the next 40 min		30 min after infusion started, his GCS improved to 11 points and was 14 after 3 h
Blixt et al. [38]	Venlafaxine	Refractory cardiovascular collapse and PEA cardiac arrest	1.5 mL/kg 20% lipid emulsion IV bolus in 60 s followed by an infusion at a rate of 0.25 mL/kg/min over 60 min		No further PEA or cardiac arrest episodes occurred

## Data Availability

Data are contained within the article.
